# Enhancement of Tomato Growth Through Rhizobacteria and Biocontrol of Associated Diseases

**DOI:** 10.3390/life15070997

**Published:** 2025-06-23

**Authors:** Hasna El hjouji, Redouan Qessaoui, Salahddine Chafiki, El Hassan Mayad, Hafsa Houmairi, Khadija Dari, Bouchaib Bencharki, Hinde Aassila

**Affiliations:** 1 Faculty of Science & Technology, Agri-Food and Health Laboratory, Hassan First University of Settat, Settat 26000, Morocco; hafsahoumairi@gmail.com (H.H.); khadija.dari@uhp.ma (K.D.); bouchaib.bencharki@uhp.ac.ma (B.B.); hind.aassila@uhp.ac.ma (H.A.); 2 Regional Center of Agricultural Research of Agadir, National Institute of Agricultural Research, Avenue Ennasr, BP415 Rabat Principale, Rabat 10090, Morocco; salahddine.chafiki@um6p.ma; 3 AgroBioSciences Plant Stress Physiology Laboratory (AgBS), Mohammed VI Polytechnic University (UM6P), Benguerir 43150, Morocco; 4 Faculty of Science, Laboratory of Biotechnology and Valorization of Natural Resources, Ibn Zohr University of Agadir, Agadir 80000, Morocco; e.mayad@uiz.ac.ma

**Keywords:** plant growth-promoting rhizobacteria, *Bacillus* spp., biocontrol agents, tomato

## Abstract

The purpose of this study was to investigate the growth-promoting effects of four rhizobacterial isolates (RS60, RS65, RS46, and RP6) isolated from the tomato rhizosphere. These isolates were screened for key plant growth-promoting rhizobacteria (PGPR) mechanisms, including ammonia production, nitrogen fixation, phosphate solubilization, indole-3-acetic acid (IAA) production, and siderophore synthesis. Their potential to enhance seed germination and tomato plant growth was investigated in controlled and greenhouse conditions. Four isolates exhibited multiple PGPR attributes, notably IAA and ammonia production as well as phosphate solubilization. The results revealed that these strains significantly enhanced tomato seed germination and shoot growth in vitro, with RS65 showing the highest germination rate (70%). However, no significant differences in early seedling responses were observed under greenhouse conditions when compared to the control. Thirty days after inoculation, greenhouse results revealed that the four studied strains significantly increased growth metrics including shoot length, number of leaves, collar diameter, and dry weight. The isolate RP6 showed a significant effect on the growth of the plant, with an average shoot length of 34.40 cm and nine leaves per plant. In vitro antagonism assays demonstrated that isolates RS60, RS65, and RP6 effectively inhibited the growth of *Botrytis cinerea*, *Alternaria alternata*, and *Oidium lycopersici*, with inhibition rates exceeding 65%. These antagonistic activities were linked to the production of hydrolytic enzymes (chitinase, cellulase, pectinase, protease), siderophores, and hydrogen cyanide (HCN). Molecular identification through 16S rRNA gene sequencing confirmed the isolates as *Bacillus cereus* (RS60), *Bacillus pumilus* (RS46), *Bacillus amyloliquefaciens* (RP6), and *Bacillus velezensis* (RS65), each showing over 97% sequence similarity with reference strains. These findings underscore the potential of the selected Bacillus spp. as promising biofertilizers and biocontrol agents for sustainable tomato cultivation and support their inclusion in integrated disease and nutrient management strategies.

## 1. Introduction

PGPR are recognized as one of the essential components of the soil microbiota associated with plant roots. These bacteria are classed as rhizosphere bacteria, and they can promote plant growth through a variety of ways. These methods include phosphate solubilization, siderophore release, nitrogen fixation, phytohormone synthesis, antifungal properties, and the induction of systemic plant resistance. These properties make PGPRs extremely useful as biofertilizers in sustainable agriculture [1]. They have emerged as potent alternatives for reducing the usage of agrochemicals such as fertilizers and pesticides, which cause contamination of soil, fruits, and vegetables. The serious threat of agrochemicals has been widely questioned by field workers, who are seriously seeking healthy alternatives to limit the use of agrochemicals.

Many studies have demonstrated that using PGPR, including biofertilizers and biopesticides, has been found to be the most effective organic alternative [2,3,4,5,6,7]. Their overall abundance in the rhizobacterial community makes them easy to use. PGPR also help to increase the seed germination, as well as plant growth and vigor of several crops [4,8,9]. Furthermore, PGPR are beneficial microorganisms that are increasingly used as sustainable alternatives to synthetic agrochemicals and can enhance plant growth by supplying essential nutrients, while also contributing to environmental sustainability and maintaining soil fertility [10,11]. Scientists have analyzed PGPR, and they have inferred that the *Bacillus* and *Pseudomonas* genera represent the core of PGPR for many crops [12,13,14,15,16,17,18,19]. Studies have reported that PGPR have a dual function. Direct action is characterized by the production of metabolites that directly affect plant growth [20]. Sultana et al. (2021) [21] showed that PGPR-produced compounds such as siderophores play a vital role in encouraging plant growth, by producing metabolites such as plant hormones, including IAA [22,23,24], gibberellic acid [25], and cytokinin [26]. PGPR are also characterized by their ability to solubilize phosphate [27,28]. Mishra et al. (2010) [29] reported that *P. fluorescens* MA-4 was the most efficient PGPR in producing ammonia and significantly increasing the biomass of geranium plant. Indirectly, these bacteria stimulate plant development by root colonization [30,31,32,33,34] and suppressing diseases caused by pathogens [35]. Samaras et al. (2021) [36] reported that *Bacillus* strains capable of colonizing plant roots colonize the whole root system [24]. These findings have positioned PGPR as sound alternatives to synthetic chemicals. The objective of this work was to investigate the influence of rhizospheric bacteria on tomato plant growth and to identify the mechanisms of action used.

## 2. Materials and Methods

### 2.1. Isolation and Preparation of Bacterial Strains

Soil samples were collected from a tomato greenhouse in Douar Ifriane (Souss-Massa, Morocco) (30°08′53.5″ N 9°36′42.4″ W) at a depth of 15–25 cm. A composite sample (~500 g) consisting of rhizosphere soil and root fragments was obtained using a zigzag method and kept at 4 °C for processing. Bacteria were isolated via standard dilution plating on nutrient agar [1], following the gentle detachment of soil particles from the root surfaces. Single colonies were purified through subculturing and maintained at −80 °C in glycerol stocks [6,37].

### 2.2. Screening of Plant Growth-Promoting Traits

Four isolates (RS60, RS65, RS46, RP6) were screened for key PGPR characteristics. Nitrogen fixation ability was checked using bromothymol blue-supplemented nitrogen-free medium [38,39]. Ammonia production was assessed in peptone water using Nessler’s reagent [40,41]. Phosphate solubilization was detected on Pikovskaya’s agar (Supplementary Materials) by observing halo formation [42]. IAA synthesis was examined by cultivating isolates in tryptophan-supplemented LB medium, followed by a Salkowski color reaction [43,44]. Siderophore production was evaluated using CAS assay with culture supernatants [45,46].

### 2.3. Germination and Growth Promotion Assays

Surface-sterilized tomato seeds (*Solanum lycopersicum*, cv. Edmundo) were soaked in bacterial suspensions (10^8^ CFU/mL) for 2 h [47,48,49]. Control seeds were treated with sterile LB medium [48]. For in vitro assays, seeds were placed on moistened sterile filter paper in Petri dishes and incubated at 25 °C. Germination percentage and shoot length were recorded after 3 days using the following formula:

$$Germination\;rate\;\left(\%\right)=\frac{Number\;of\;germinated\;seeds}{Total\;number\;of\;seeds\;}\times 100$$

Shoot length was measured using a flexible thread along the seedling axis and then transferred to a ruler.

### 2.4. Greenhouse Evaluation

Disinfected seeds were sown in sterilized 28-well trays containing a peat–sand mix (2:1 *v*/*v*) [33]. Trays were covered with foil for initial germination. The daily assessment of seed germination and shoot length was conducted during the first week to calculate the germination percentage and shoot length [50]. Environmental conditions were maintained at 28 °C with 60–70% humidity. After 10 days, uniform seedlings were transplanted to pots filled with sterilized substrate. Plants were grown under greenhouse conditions (14 h photoperiod, 18–26 °C), with weekly NPK fertilization. Growth parameters (shoot/root length, leaf number, collar diameter, and biomass) were recorded after 30 days [51,52].

### 2.5. In Vitro Antagonism Against Fungal Pathogens

Pathogenic fungi (*Botrytis cinerea*, *Alternaria alternata*, and *Oidium lycopersici*) were isolated from infected tomato tissues. Dual culture assays were performed by placing fungal plugs at the center of PDA plates and inoculating bacterial strains at three equidistant points [53,54,55]. Plates were incubated at 25 °C in the dark for 5–7 days [56]. Mycelial inhibition percentage was calculated using the following formula [57]:

MIP = [(r1 − r2)/r1] × 100

where r1 represents the fungus’s radial growth in the control, whereas r2 represents the fungus’s radial growth in direct confrontation with the bacterial isolate.

### 2.6. Analysis of Antagonistic Mechanisms

Selected bacterial strains were evaluated for the production of HCN and hydrolytic enzymes. HCN production was tested using picrate-saturated filter papers [58,59]. Enzymatic assays included detection lipase (Tween 80 agar) [60,61], protease (skim milk agar) [6,62,63,64], chitinase (colloidal chitin agar) [65,66], cellulase (CMC agar) [67,68], pectinase (pectin agar) [6,68,69,70], and glucanase (β-glucan agar) [68,71,72]. Activity was determined by halo formation after specific staining when applicable.

### 2.7. Molecular Characterization

DNA was extracted and amplified using universal primers targeting the 16S rRNA gene [6]. Amplicons (~1500 bp) were sequenced and compared to GenBank entries via BLAST. Phylogenetic trees were constructed using UPGMA clustering with Bionumerics v7.6 [6,73,74]. Isolates were deposited under accession numbers PV489846 (RP6), PV490961 (RS46), and PV523529 (RS60).

### 2.8. Statistical Analysis

Data were analyzed using one-way ANOVA (*p* < 0.05) via IBM SPSS Statistics 26. Means were compared using the Student–Newman–Keuls post hoc test. All experiments were conducted in triplicate.

## 3. Results

### 3.1. Isolation and Purification of Bacterial Isolates

A total of four bacterial isolates were obtained on the nutrient agar medium, exhibiting a variety of colors and morphologies. Of these, three isolates were derived from the rhizosphere (RS) and one from the rhizoplane (RP).

### 3.2. In Vitro Results of PGPR Isolates

The isolates were assessed for their plant growth-promoting (PGP) potential based on their ability to solubilize phosphate; produce indole-3-acetic acid (IAA), siderophores, and ammonia; and fix atmospheric nitrogen. The results indicated that all isolates exhibited phosphate solubilization and ammonia production. Phosphate solubilization was evidenced by the formation of clear halos around the bacterial colonies. Three isolates (RP6, RS46, and RS60) were able to synthesize IAA, as confirmed by the development of a pink/red coloration upon reaction with Salkowski’s reagent. Among all tested strains, only RS46 demonstrated siderophore production, indicated by the appearance of an orange halo on the Chrome Azurol S (CAS) medium. Additionally, none of the isolates exhibited nitrogen fixation activity (Table 1).

### 3.3. In Vitro Effect of Isolates on Seed Germination and Shoot Length

**Figure 1:**
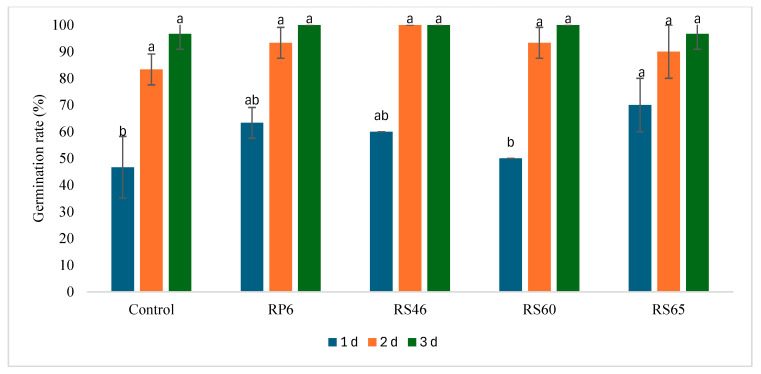
Effect of isolates on seed germination compared to the control. Bars with same letters are not significantly different at *p* < 0.05, based on Student–Newman–Keuls test.

**Figure 2:**
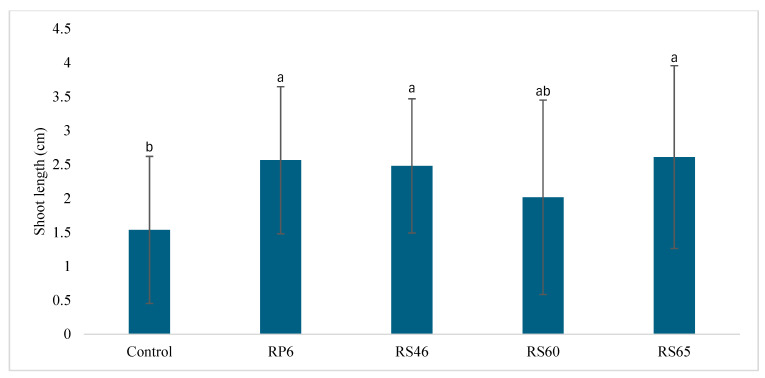
Effect of isolates on shoot length compared to the control. Bars with same letters are not significantly different at *p* < 0.05, based on Student–Newman–Keuls test.

Tomato seeds containing the four isolates were distinguished based on their potential efficacy for seed germination and shoot growth under Petri dish conditions. Among the four tested strains, RS65 was characterized by the highest and most significant effect on germination percentage (70%) compared to the control (47%) after 24 h of incubation (Figure 1). The results show that this isolate stimulates germination. Three days after germination, the shoot height was enhanced significantly by three isolates (RP6, RS46, and RS65), ranging from 2.5 cm to 2.6 cm by RP6 and RS65, respectively, when compared to the control (1.5 cm) (Figure 2).

### 3.4. Greenhouse Seed Germination and Seedling Height Measurement

**Figure 3:**
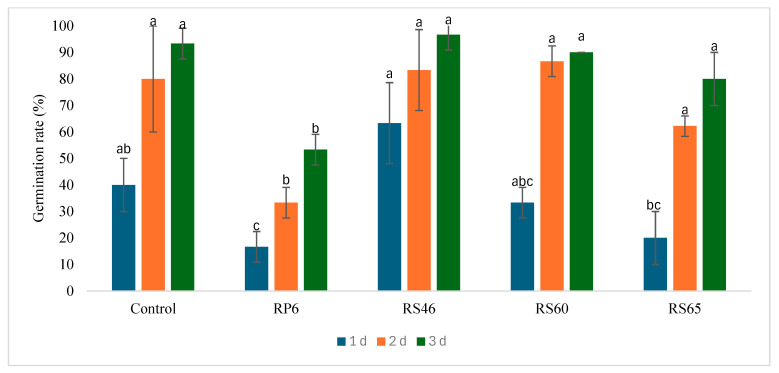
Effect of isolates on seed germination under greenhouse conditions. Bars with same letters are not significantly different at *p* < 0.05, based on Student–Newman–Keuls test.

**Figure 4:**
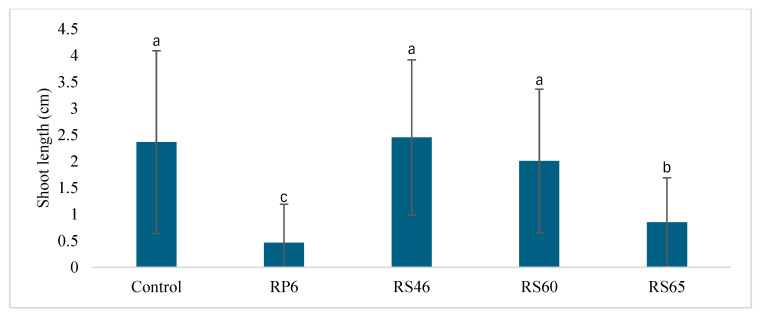
Effect of isolates on shoot height after germination in vivo. Bars with same letters are not significantly different at *p* < 0.05, based on Student–Newman–Keuls test.

The four strains were tested for seed germination under greenhouse conditions, and they did not show any significant differences from the control during the three days of germination (Figure 3). The same is true for shoot length. A statistical analysis revealed no significant increase in the shoot length three days after germination compared to the control (Figure 4).

### 3.5. In Vivo Effect of Isolates on Plant Growth

**Figure 5:**
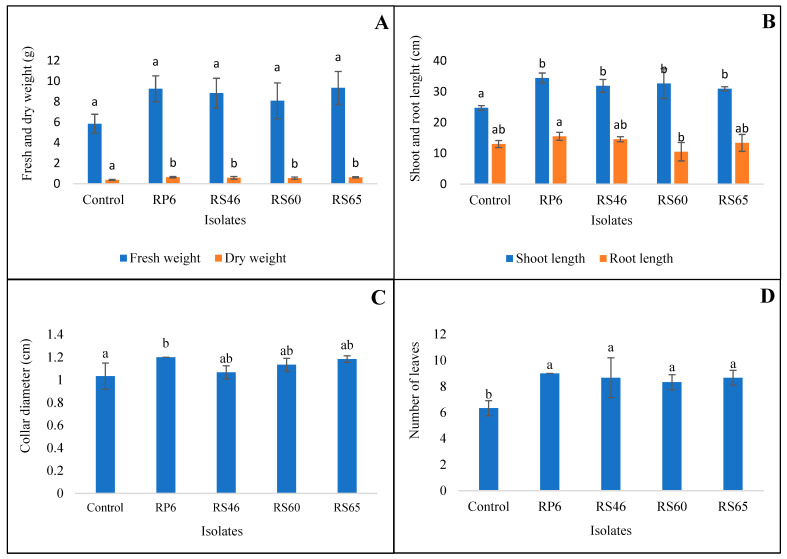
Comparison of plant growth among *Bacillus* spp.-treated plants 30 days after transplantation. Fresh and dry weights (**A**), shoot and root length (**B**), collar diameter (**C**), and number of leaves/plant (**D**). Bars with same letters are not significantly different at *p* < 0.05, based on Student–Newman–Keuls test.

The effect of the isolates on the growth parameters of the tomato plants was observed under greenhouse conditions for 30 days after transplantation. The following variables were measured: plant length, root length, number of leaves (aerial region), and collar diameter. The findings of this study demonstrated that all bacterial strains significantly increased the length and number of leaves and root length (*p* < 0.01) compared to the control. A substantial length and quantity of leaves were obtained with RP6, with a length of 34.40 cm and nine leaves per plant, compared to the control (Figure 5).

For the collar diameter, the isolate RP6 showed a significant effect compared to the control. However, no significant difference was shown for the root length (Figure 5).

For the fresh and dry weights, the results of this study showed that all bacteria significantly increased (*p* < 0.01) the dry weight of the tomato plants 30 days after transplantation. The most significant increase in dry weight was obtained with RP6 (0.64 cm) compared to the control (Figure 5).

### 3.6. Biocontrol Effect of Selected Isolates

**Figure 6:**
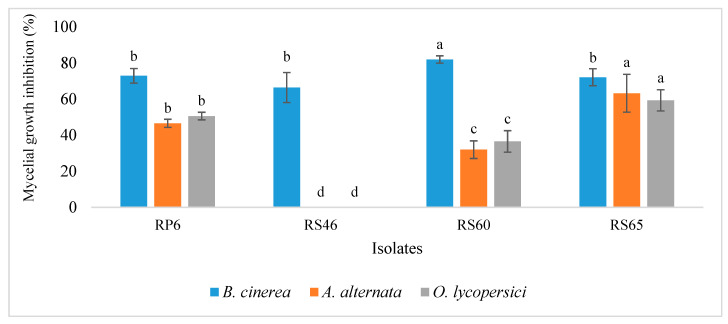
Effect of isolates against plant pathogens. Bars with same letters are not significantly different at *p* < 0.05, based on Student–Newman–Keuls test.

**Figure 7:**
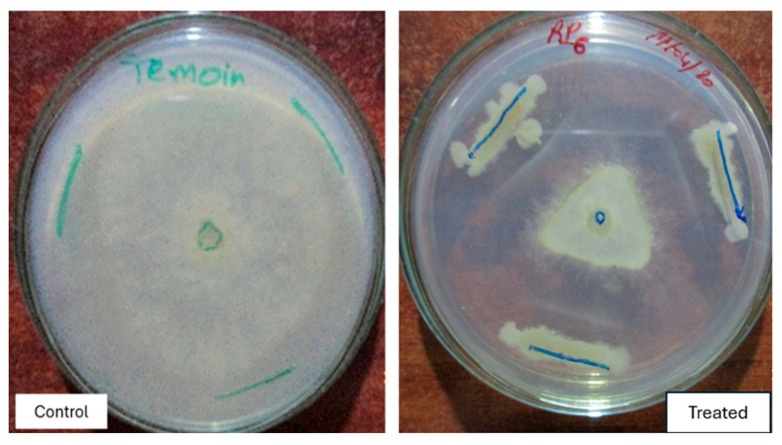
Antagonistic effect of the isolates against *B. cinerea*.

The isolates were also tested to evaluate their antagonistic activity against plant pathogens. The results show that all four isolates exhibited significant inhibition against *Botrytis cinerea*. Statistical analysis indicated that all isolates showed inhibition above 65%, with RS60 demonstrating the highest inhibition at 80%. These results indicate a strong biopesticide potential against *B. cinerea* (Figure 6 and Figure 7).

Regarding *A*. *alternata*, the results showed that isolate RS65 exhibited significant inhibition, exceeding 63%. In contrast, RS60 and RP6 showed inhibition rates ranging from 31% to 46%. However, RS46 had no effect on the development of *A. alternata* (Figure 6). For the effect of isolates against *O. lycopersici*, the results showed that RS65 exhibited significant inhibition, exceeding 59%. RS60 and RP6 showed inhibition rates ranging from 36% to 50%. However, RS46 showed no effect on *O. lycopersici* (Figure 6). This inhibition manifested as a reduction in the mycelial growth of the pathogens treated with the bacterial isolates compared to the control. These results indicate a strong biopesticide potential of the three isolates (RS65, RS60, and RP6) against the three pathogens *B. cinerea*, *A. alternata*, and *O. lycopersici*.

### 3.7. Antagonism Mechanism

The results showed that the four isolates exhibited significant enzymatic activity (Table 2). In particular, the isolates RS65 and RP6 showed activity for all the targeted enzymes, except for lipolytic activity, for which the two isolates did not show any activity, although they produced a low level of chitinase. In contrast, the other isolates lacked activity for two enzymes, glucanase and lipolytic, for RS60 and HCN, and pectinase for RS46 (Table 2).

### 3.8. Molecular Identification

**Figure 8:**
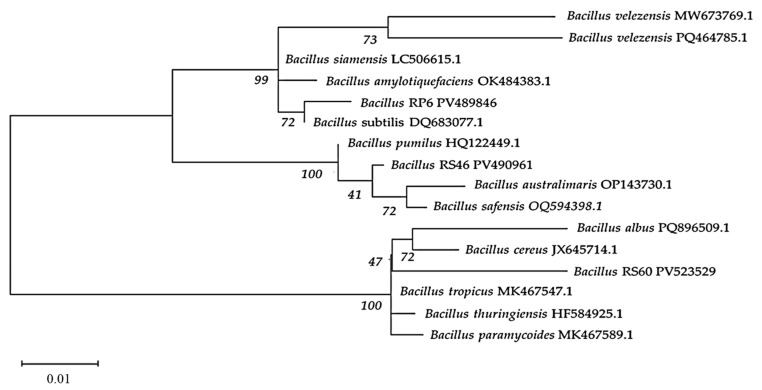
Phylogenetic tree of strains RP6 (PV489846), RS46 (PV490961), and RS60 (PV523529) constructed based on alignment of partial sequences of 16S ribosomal RNA gene using NCBI platform, by applying the neighbor-joining method with bootstrap support.

The isolate RS65 was identified as *Bacillus velezensis* RS65. For the other three isolates, the results showed that they clustered with several strains of the *Bacillus* genus (Figure 8), displaying significant genetic proximity supported by high bootstrap values (greater than 97%), which indicates strong confidence in these phylogenetic relationships. The sequences of each isolate were submitted to the GenBank reference database under the accession numbers PV489846, PV490961, and PV523529 for RP6, RS46, and RS60, respectively, and were identified as follows:

RS60: *Bacillus cereus* JX645714 (97.83% genetic similarity);

RS46: *Bacillus pumilus* HQ122449.1 (99.45% genetic similarity);

RP6: *Bacillus amyloliquefaciens* OK484383.1 (99.06% genetic similarity).

The presence of *Bacillus* strains closely related to these isolates suggests potential phenotypic similarities, particularly in their ability to produce enzymes and secondary metabolites with biopesticide activity.

## 4. Discussion and Conclusions

The plant growth-promoting rhizobacterial strains have been well documented across various crops [75]. Bacterial inoculants have been shown to enhance tomato plant growth and development through several mechanisms, including increased germination rates, improved tomato seedling emergence, and enhanced protection against phytopathogens [76,77]. This study additionally confirmed the effect of PGPRs on tomato plant growth and demonstrated that four bacterial strains significantly stimulate plant growth under greenhouse conditions compared to the control. The bacterial strain showed a significant effect on seed germination rate in vitro conditions. However, under greenhouse conditions, the effect on germination and emergence during the first three days after sowing was not significant. This difference highlights the difference between in vitro and in vivo conditions. Under greenhouse conditions, the bacteria may require more time to colonize the root system, and environmental factors may also influence their activity. Additionally, the presence of other soil microorganisms can either inhibit or enhance the effect of the inoculated strains. In the same context, Qessaoui et al. (2019) [4] showed that strains of *Pseudomonas* spp. (Q6B, Q14B, Q7B, Q1B, and Q13B) significantly promoted plant growth. All of these five strains significantly promoted plant length and increased the size of the collar diameter and the leaf number compared to the control. Gholami et al. (2009) [78] reported that seed inoculation with six bacterial strains (*P. putida* strain R-168, *P. fluorescens* strain R-93, *P. fluorescens* DSM 50090, *P. putida* DSM 291, *A. lipoferum* DSM 1691, and *A. brasilense* DSM 1690) significantly enhanced the seed germination and seedling vigor of maize. Furthermore, the presence of these organisms has been shown to result in a substantial increase in the dry weight of leaves and shoots, as well as the augmentation of leaf surface area, in both sterile and non-sterile soil. Chabbi et al. (2024) [9] confirmed the growth-promoting potential of rhizobacteria for plants. Their findings demonstrated that *Leucobacter aridicollis* sp1 significantly enhanced the germination rate by 95.83% and increased the radicle length to 2.71 cm compared to the control (1.60 cm). Under greenhouse conditions, the plant height showed a marked increase of 42.07% and 39.99%, with *L. aridicollis* sp1 and sp2, respectively. Furthermore, *Brevundimonas naejangsanensis* sp3 and *L. aridicollis* sp1 led to significant improvements in the collar diameter, with increases of 41.56% and 41.21%, followed by *L. aridicollis* sp2 and *Staphylococcus saprophyticus* (38.68% and 22.79%, respectively). Notably, *L. aridicollis* sp1 also significantly enhanced the number of branches per plant, reaching an average of 12 branches per plant compared to the control. The plant growth-promoting effects of these PGPR strains are primarily attributed to their abilities to solubilize phosphate, produce auxins and siderophores, and fix atmospheric nitrogen [79]. In this study, four bacterial strains exhibited phosphate-solubilizing activity and ammonia production. Among these, the isolates RP6, RS46, and RS60 were found to produce indole-3-acetic acid (IAA). Notably, only the RS46 isolate demonstrated the ability to produce siderophores. However, none of the tested strains exhibited nitrogen fixation capacity. Many studies demonstrated that growth stimulation mechanisms, phytohormone production, phosphate solubilization, ammonia production, and root plant colonization are the most efficacious mechanisms that explain PGPR effects [17,80,81]. Phosphate solubilization by plant growth-promoting rhizobacteria (PGPRs) is primarily attributed to the production of various organic acids and enzymes, which convert the insoluble forms of phosphate into soluble forms that are readily assimilable by plants [82,83,84,85,86]. The key organic acids involved in this process are gluconic, tartaric, and oxalic acids [82,83]. In addition, PGPRs commonly produce indole-3-acetic acid (IAA), the most important auxin synthesized by bacteria, plants, and fungi [87]. IAA plays a critical role in initiating the formation of roots, leaves, and flowers, and is central to processes such as cell division, elongation, fruit development, and senescence [88]. Another important trait of PGPRs is the production of siderophores [89]. In addition to the PGPR effect, these strains also have a potential antagonist effect against plant pathogens. The results of this study indicate that three bacterial isolates (RS65, RS60, and RP6) were effective against the phytopathogens *B. cinerea*, *A. alternata*, and *Oidium lycopersici*. All three isolates exhibited strong inhibitory activity against *B. cinerea*, with inhibition rates exceeding 65%. In the case of *A. alternata*, RS65 showed the highest inhibition (>63%), while RS60 and RP6 displayed moderate inhibition levels ranging from 31% to 46%. Similarly, RS65 demonstrated notable inhibitory activity against *O. lycopersici* (>59%), whereas RS60 and RP6 showed moderate inhibition between 36% and 50%. The antagonistic effects of these bacterial strains are generally attributed to their ability to produce antimicrobial compounds and hydrolytic enzymes such as chitinase, pectinase, hydrogen cyanide (HCN), and siderophores and induced systemic resistance (ISR). The analysis of antagonistic mechanisms revealed that all four isolates demonstrated notable enzymatic activity [90]. Specifically, the isolates RS65 and RP6 showed activity for all tested enzymes, except lipolytic enzymes. In the same context, many studies showed the importance of PGPRs in controlling plant diseases. Ramette et al. (2003) [91] reported that the microbial production of HCN is an important antifungal trait in the control of root-infecting fungi. In a similar context, Kumari and Khanna (2014) [92] reported that the plant growth-promoting rhizobacterial isolate (15B) significantly inhibited the growth of *F. oxysporum* f.sp. ciceri by producing volatile organic compounds (VOCs), resulting in 64.2% inhibition compared to controls. Certain enzyme-producing bacteria can destroy the oospores of phytopathogenic fungi [93] and influence the spore germination and germ-tube elongation of phytopathogenic fungi [94,95]. Qessaoui et al. (2021) [5] reported that five *Pseudomonas* strains (Q6B, Q13B, Q7B, Q14B, and Q1B) produced antifungal metabolites—including siderophores, hydrogen cyanide (HCN), and hydrolytic enzymes—resulting in the significant inhibition of fungal growth, with inhibition levels ranging from 65% to 73%. These isolates were effective in suppressing fungal development on both fruits and leaves. Elsharkawy et al. (2022) [96] showed that four *Pseudomonas* isolates effectively induce systemic resistance in rice plants against sheath blight, enhancing the production of peroxidase and polyphenol oxidase enzymes and the expression of the *phenylalanine ammonia lyase* (*PAL*) and *NPR1* genes, which could be involved in disease incidence reduction. The results of this study demonstrated that these isolates were identified as belonging to the *Bacillus* genus, with a high similarity rate. RS60 is closely related to *Bacillus cereus*, RS46 is closely related to *Bacillus pumilus*, and RP6 is closely related to *Bacillus amyloliquefaciens*. These species are recognized for their biopesticide properties [97,98,99,100], suggesting that these strains may exhibit similar traits. These findings support the hypothesis that *Bacillus* spp. could be effectively integrated into disease management programs for the sustainable control of tomato pathogens. *Bacillus* is a versatile bacterial genus known for its ability to thrive in diverse environments and its resilience to various abiotic stresses. In addition to its biocontrol properties, *Bacillus* exhibits plant growth-promoting rhizobacterial traits. Several strains of *Bacillus pumilus*, such as *B. pumilus* LZP02 and EU927414, have been identified as effective plant growth-promoting bacteria (PGPBs). These strains are known to produce a wide array of phytohormones and other bioactive compounds that contribute to plant development and stress tolerance [101]. As a result, the four selected *Bacillus* strains can be classified as both plant growth-promoting rhizobacteria (PGPRs) and effective biocontrol agents. Their dual functionality highlights their potential as biological fertilizers and eco-friendly alternatives to chemical pesticides, which are often detrimental to environmental and human health. The application of such beneficial bacteria may significantly reduce, or even eliminate, the need for synthetic fertilizers, thus contributing to more sustainable and environmentally responsible farming practices [3,88]. This study presents several limitations related to both the application of bacterial strains against phytopathogens and their plant growth-promoting (PGPR) effects. First, the antagonistic activity of the strains was assessed in vitro, which may not fully reflect their behavior in natural soil conditions, where environmental variables and microbial interactions are more complex. Second, soil microbiota interactions were not extensively optimized or evaluated. To overcome these limitations, further research should focus on evaluating the antagonistic effects of the selected *Bacillus* strains under greenhouse conditions and exploring their potential as candidate biofertilizers across a wider range of plant species to minimize disruption to ecosystem structure. Future investigations are also recommended to include both biochemical and molecular approaches. Biochemically, emphasis should be placed on elucidating the mechanisms and pathways involved in auxin biosynthesis [102], siderophore production, and phosphate solubilization [103]. These insights will be critical for understanding the full potential of these strains in sustainable agriculture.

## Figures and Tables

**Table 1:** PGPR mechanisms of four isolates.

	Nitrogen Fixation	Ammonia Production *	Phosphate Solubilization	IAA Production *	Siderophore Production *
RP6	-	++	+	++	-
RS46	-	++	+	++	+
RS60	-	++	+	++	-
RS65	-	++	+	-	-

Note: * no production is denoted by (-) moderate production by denoted by (+), high production by denoted by (++).

**Table 2:** Enzymatic activity of four selected isolates.

	Production of Cyanide HCN *	Lipolytic Activity	Proteolytic Activity	Chitinolytic Activity	Cellulase Production	Pectinase Production	Glucanase Production
RS60	++	-	++	++	++	+++	-
RP6	++	-	++	+	++	+++	+++
RS65	++	-	++	+	++	+++	++
RS46	-	++	++	+	++	-	+

Note: * no production is denoted by (-), low production denoted by (+), medium production by denoted by (++), high production by denoted by (+++). The level of HCN production is indicated by the color intensity of the filter paper, which changes from orange to brown (orange indicating low production; brown indicating high production).

## Data Availability

The original contributions presented in the study are included in the article/Supplementary Materials, further inquiries can be directed to the corresponding author.

## Supplementary Materials

The following supporting information can be downloaded at: https://www.mdpi.com/article/10.3390/life15070997/s1, Composition of culture media.
