# Supplementary material for: Enhancement of Tomato Growth Through Rhizobacteria and Biocontrol of Associated Diseases
Source: Life (Basel). 2025 Jun 23;15(7):997. doi: 10.3390/life15070997 (PMC12298581; doi:10.3390/life15070997)
Supplement: Supplementary file 1 [file life-15-00997-s001.zip › life-3653430-supplementary.pdf]

Supplementary material

Medium	Composition (1L)
NF-MM medium	Malic acid (5 g), agar (15 g), K2HPO4 (0.5 g), KOH (4 g), CaCl2 (0.02 g), NaCl (0.1 g), MgSO4 · 7H2O (0.2 g), ZnSO4 7H2O (0.24 g), H3BO3 (0.280 g), CuSO4 · 5H2O (0.008 g), FeSO4 · 7H2O (0.01 g), Na2MoO4 · 2H2O (0.2 g), MnSO4 · 5H2O (0.235 g), and bromothymol blue (5% solution) (2 mL), adjusted to pH 7.0.
The PVK medium composition	Yeast extract 0.50 g, dextrose 10.00 g, $Ca_3(PO_4)_2$ 5.00 g, $(NH_4)_2SO_4$ 0.500 g, KCl 0.20 g, $MgSO_4$ 0.10 g, $MnSO_4$ 0.0001 g, $FeSO_4$ 0.0001 g, and agar 15.00 g.
LPGA medium	7g yeast extract, 7g peptone, 7g glucose, and 18 g agar per liter
the peptone agar medium	10 ml Tween80, 10 g peptone, 5 g NaCl, 0.1 g $CaCl_2$, and 15 g agar
milk agar medium	100 g skimmed milk powder, 5 g peptone
colloidal chitin agar medium	0.07% K_2HPO_4, 0.05% $MgSO_4$, 0.03% KH_2PO_4, 0.001% $FeSO_4$, 0.03% KH_2PO_4, 2% colloidal chitin and 1.5% agar
carboxymethyl cellulose agar	10 g of carboxymethyl cellulose (sodium salt), 1 g K_2HPO_4, 0.2 g $MgSO_4{\cdot}7H_2O$, 1 g NH_4NO_3, 0.05 g $FeCl_3{\cdot}6H_2O$, 0.02 g $CaCl_2$, and 20 g agar
